# Supplementary material for: The Construction and Exploration of a Comprehensive MicroRNA Centered Regulatory Network in Foxtail Millet (Setaria italica L.)
Source: Front Plant Sci. 2022 May 6;13:848474. doi: 10.3389/fpls.2022.848474 (PMC9121102; doi:10.3389/fpls.2022.848474)
Supplement: Supplementary file 2 [file Data_Sheet_2.zip › Sit-miR394_Seita.9G344400.1_495_TPlot.pdf]

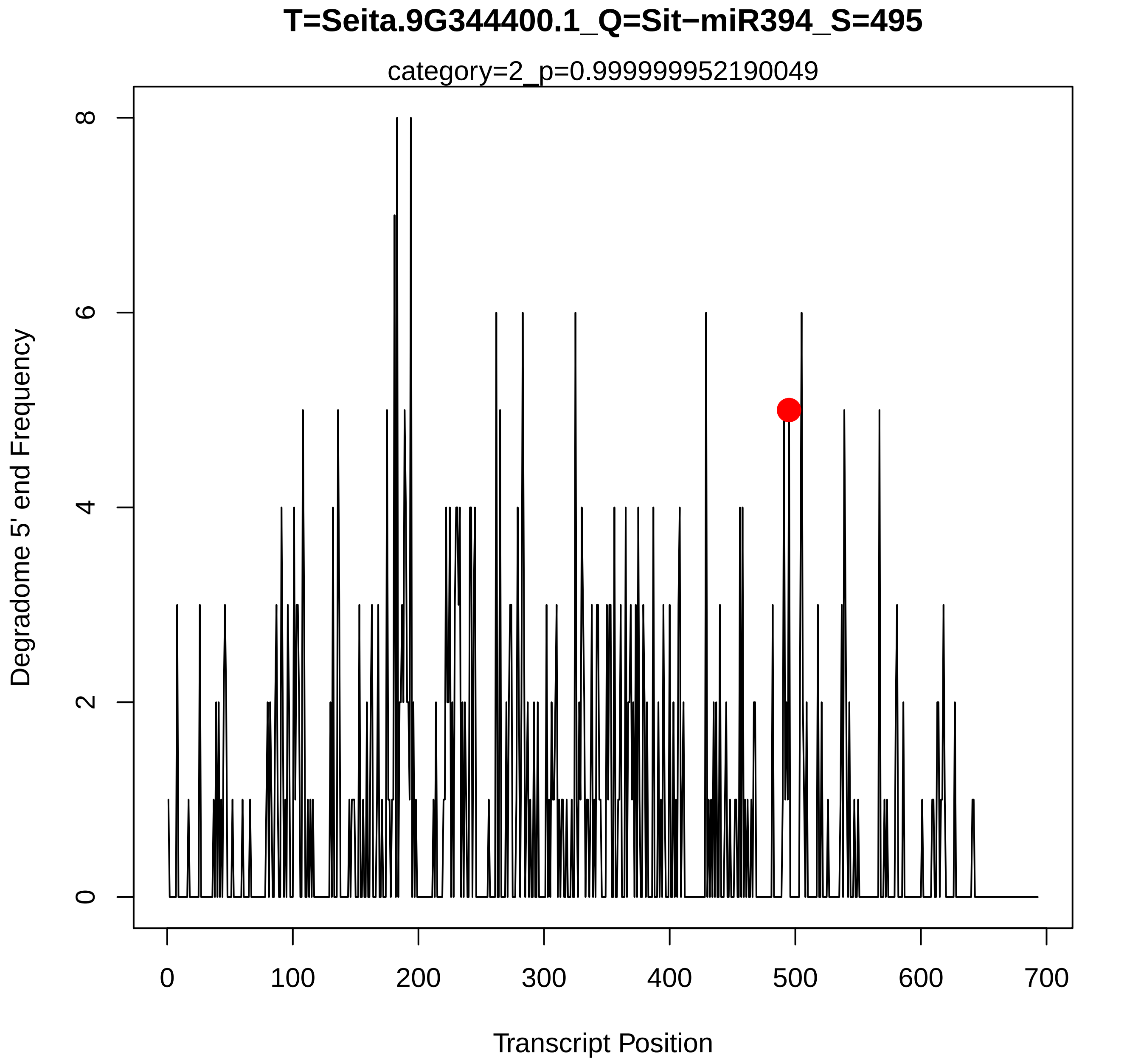
T=Seita.9G344400.1_Q=Sit−miR394_S=495
category=2_p=0.999999952190049
Degradome 5' end Frequency
0
2
4
6
8
0
100
200
300
400
500
600
700
Transcript Position
